# *Brachythecium wangianum* (Brachytheciaceae, Bryophyta), a new species from Northern China and Southern Russia

**DOI:** 10.3897/phytokeys.278.205832

**Published:** 2026-08-20

**Authors:** Xiao-Tong Peng, Michael S. Ignatov, Qian-Nan Gu, Hong-Zhu Liang, Cai-Xia Chen, Yu-Ting Wang, Min Li

**Affiliations:** 1 College of Life Sciences, Hebei Normal University, Shijiazhuang 050024, China College of Life Sciences, Hebei Normal University Shijiazhuang China; 2 Tsitsin Main Botanical Garden, Moscow 127276, Russia Tsitsin Main Botanical Garden Moscow Russia; 3 Wulingshan National Nature Reserve, Chengde 067300, China Wulingshan National Nature Reserve Chengde China

**Keywords:** Molecular phylogeny, morphology, moss, nrITS

## Abstract

*Brachythecium wangianum* (Brachytheciaceae), a new moss species from northern China and southern Russia, is described and illustrated. Combining morphological observations and phylogenetic analyses, this study clarifies the systematic placement of Chinese and Russian materials of this taxon, provides a full morphological description, and distinguishes it from other morphologically similar species of *Brachythecium*. Special attention is paid to comparison with the closely related congeners *B. laetum* (Brid.) Schimp. and *B. boreale* Ignatov, as well as the widespread and highly polymorphic *B. salebrosum* (Hoffm. ex F. Weber & D. Mohr) Schimp. and *B. rotaeanum* De Not. Phylogenetic analysis based on nrITS sequences strongly supports *B. wangianum* as a distinct species, sister to *B. laetum* (Brid.) Schimp. Morphologically, *B. wangianum* differs from *B. laetum* in having a longer, more slender leaf acumen, longer median laminal cells, and an autoicous sexual condition. Combined with morphological and phylogenetic evidence, the taxonomic status of *B. wangianum* is established, and its geographical distribution delineated. Detailed micrographs are also provided. The discovery of this new species enriches the species diversity of *Brachythecium* in China and Russia, and provides valuable data for future studies on the phylogeny and biogeography of Brachytheciaceae in temperate regions.

## Introduction

Brachytheciaceae are one of the most taxonomically challenging groups amongst pleurocarpous mosses (Huttunen and Ignatov 2004). By combining morphological and molecular data, Ignatov and Huttunen (2002) clarified the phylogenetic relationships within the family, refined taxonomic boundaries, and improved its classification. Consequently, the family currently comprises approximately 48 genera and 560 species worldwide (Li et al. 2026).

*Brachythecium*, established by Schimper (1853), is one of the largest and most taxonomically complex genera in the family, with approximately 150 species distributed primarily in temperate and boreal regions (Frey and Stech 2009). According to the concept of *Brachythecium* s.str. proposed by Ignatov and Huttunen (2002), 30 species occur in China, eight of which are endemic: *Brachythecium bodinieri* Cardot & Thér., *B. coarctum* (Müll. Hal.) Ignatov & Huttunen, *B. formosanum* Takaki, *B. homocladum* Müll. Hal., *B. perscabrum* Broth., *B. pinnirameum* Müll. Hal., *B. procumbens* (Mitt.) A. Jaeger, and *B. sinense* (Broth.) Y. Huan Wu & W. Sheng (Li et al. 2026).

The authors have long been engaged in the systematics of Brachytheciaceae. During long-term taxonomic studies, a group of specimens collected from various provinces of China showed high sequence similarity to those identified from Russia as *Brachythecium aff. laetum* (Brid.) Schimp. (GenBank MW027557, MW027560; Fedosov et al. (2022)). A previous study noted the close morphological and phylogenetic affinity of this entity to *B. laetum*, while also recognising certain distinguishing features (Fedosov et al. 2022).

The aim of the study is to elucidate the systematic position of these Chinese specimens through both morphological examination and phylogenetic analysis, to provide a detailed description, and to distinguish them from other similar species of *Brachythecium*. Particular attention is given to comparisons with the closely related congeners *B. laetum* (Brid.) Schimp. and *B. boreale* Ignatov, as well as the widespread and highly polymorphic *B. salebrosum* (Hoffm. ex F. Weber & D. Mohr) Schimp., *B. rotaeanum* De Not., and morphologically similar *B. falcatum* (Grout) H.A. Crum.

## Materials and methods

### Morphological observations and measurements

**Figure 1.**
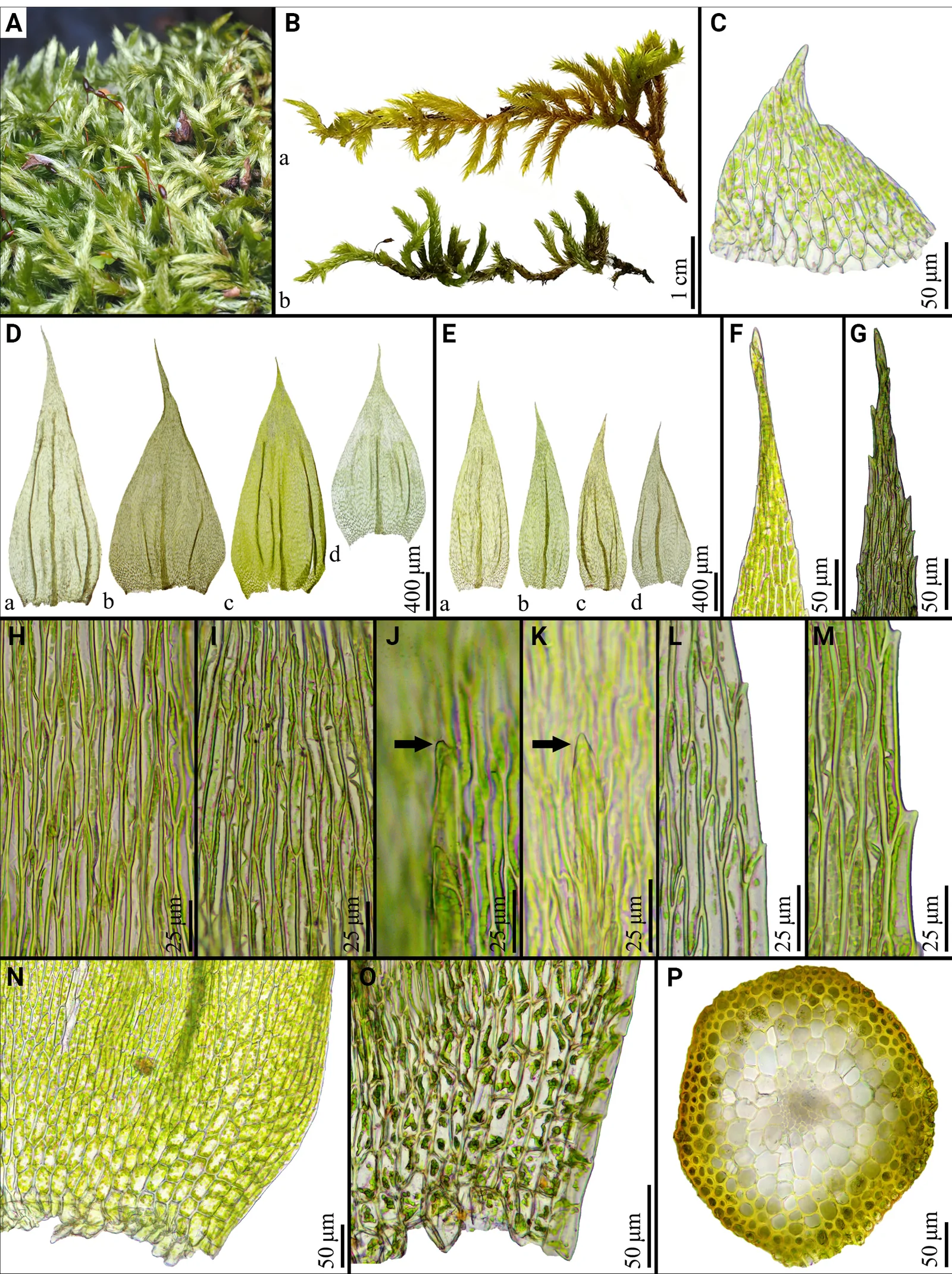
*Brachythecium wangianum* Min Li, X.T. Peng & Ignatov. **A**. Population; **B**. Plants; **C**. Pseudoparaphyllia; **D**. Stem leaves; **E**. Branch leaves; **F**. Tip of stem leaf; **G**. Tip of branch leaf; **H**. Middle cell of stem leaf; **I**. Middle cell of branch leaf; **J**. Terminal spine of stem leaf; **K**. Terminal spine of branch leaf; **L**. Middle margin of stem leaf; **M**. Middle margin of branch leaf; **N**. Alar cells of stem leaf; **O**. Alar cells of branch leaf; **P**. Transverse section of the stem (A, B, Cb, Dc, F, N and P from Holotype of *M. Li* & *X.T. Peng 20250918-66*, HBNU!; Ca and Da from *M. Li & M. Sulayman 2024050413*, HBNU!; Db, Ed and K from *M. Li 2014080301*, HBNU!; Eb, G, I, L and M from *M. Li 20200902233*, HBNU!; Ea, Ec, H, J and O from *M. Li 20200903049*, HBNU!; Dd from *M. Li 2024080343*, HBNU!).

**Figure 2.**
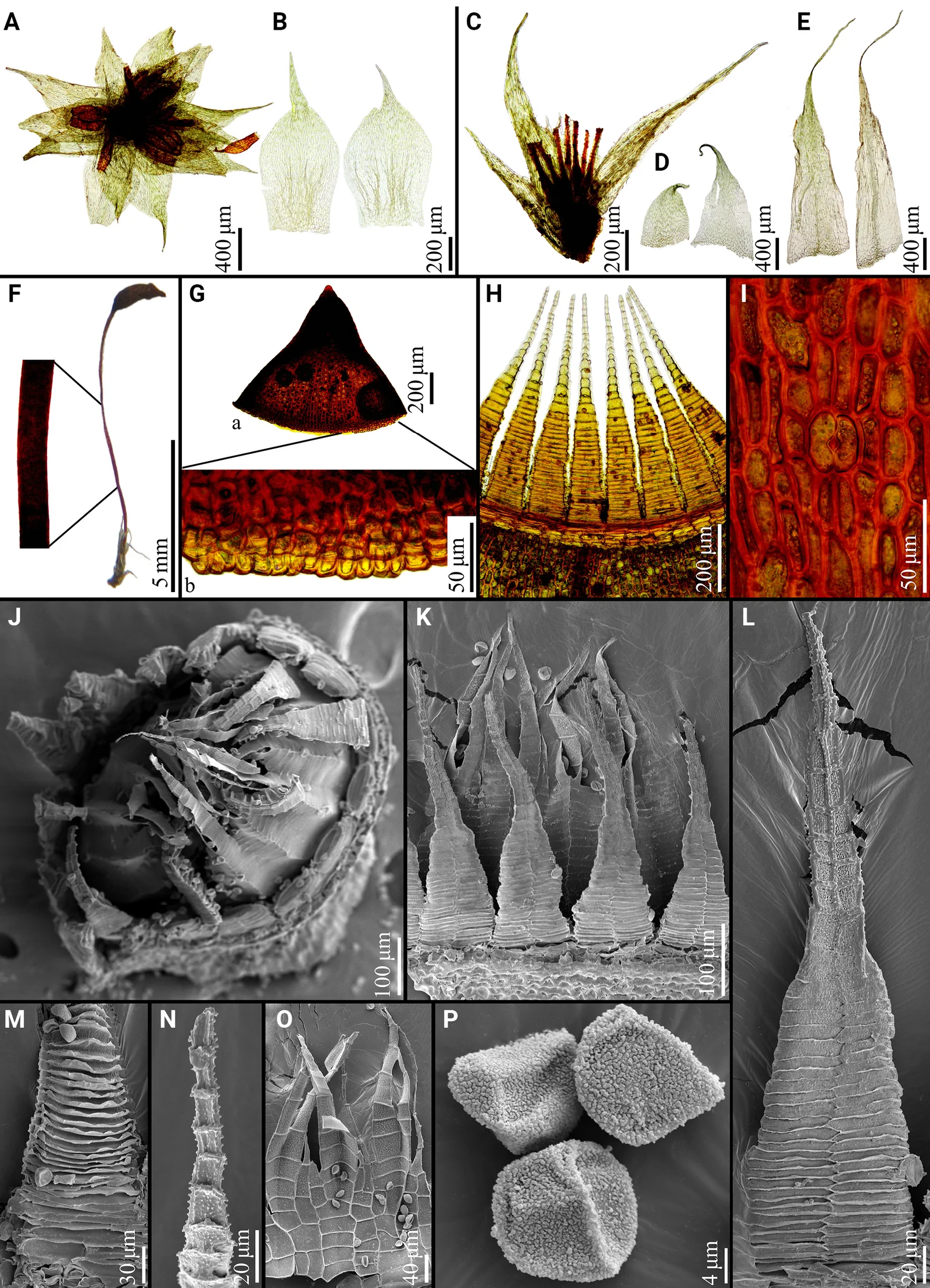
*Brachythecium wangianum* Min Li, X.T. Peng & Ignatov. **A**. Perigonium; **B**. Perigonial leaves; **C**. Perichaetium; **D**. Outer perichaetial leaves; **E**. Inner perichaetial leaves; **F**. Sporophyte; **G**. Operculum; **H**. Exostome; **I**. Stoma; **J**. Peristome; **K**. Peristome; **L**. Outside teeth; **M**. Outer surface of lower tooth; **N**. Upper part of teeth from inside; **O**. Endostome; **P**. Spores (All from Holotype *M. Li* & *X.T. Pen*g *20250918-6*6, HBNU!).

Micromorphological features were examined under a Nikon Eclipse E200 light microscope. High-resolution images of leaves and cells were captured with a Jiangnan Yongxin DC6000 digital camera and subsequently focus-stacked automatically using ImageView x64 (v. 4.12.26221.20240804). Scanning electron microscopy (SEM) was performed on a Zeiss EVO18 for ultrastructural details. Final plates were assembled using Adobe Photoshop CC 2018 (Figs 1, 2).

Morphometric measurements and statistical analyses were performed on the newly described species. For each specimen, three well-developed and intact individuals were selected, and three leaves were sampled from the mid-stem and mid-branch regions, respectively. The length and width of leaves and laminal cells, as well as the length of the costa, were measured using ImageJ (Rasband 1997–2018).

### Molecular methods and phylogenetic analyses

Total genomic DNA was extracted using a modified CTAB protocol (Li et al. 2013). The nuclear ribosomal internal transcribed spacer region (ITS1-5.8S-ITS2) was amplified following the PCR conditions described by Li et al. (2014). PCR products were purified and sequenced by Sangon Biotech (Shanghai, China). Sequences were edited and assembled in SeqMan (Swindell and Plasterer 1997) to obtain the complete ITS region.

**Figure 3.**
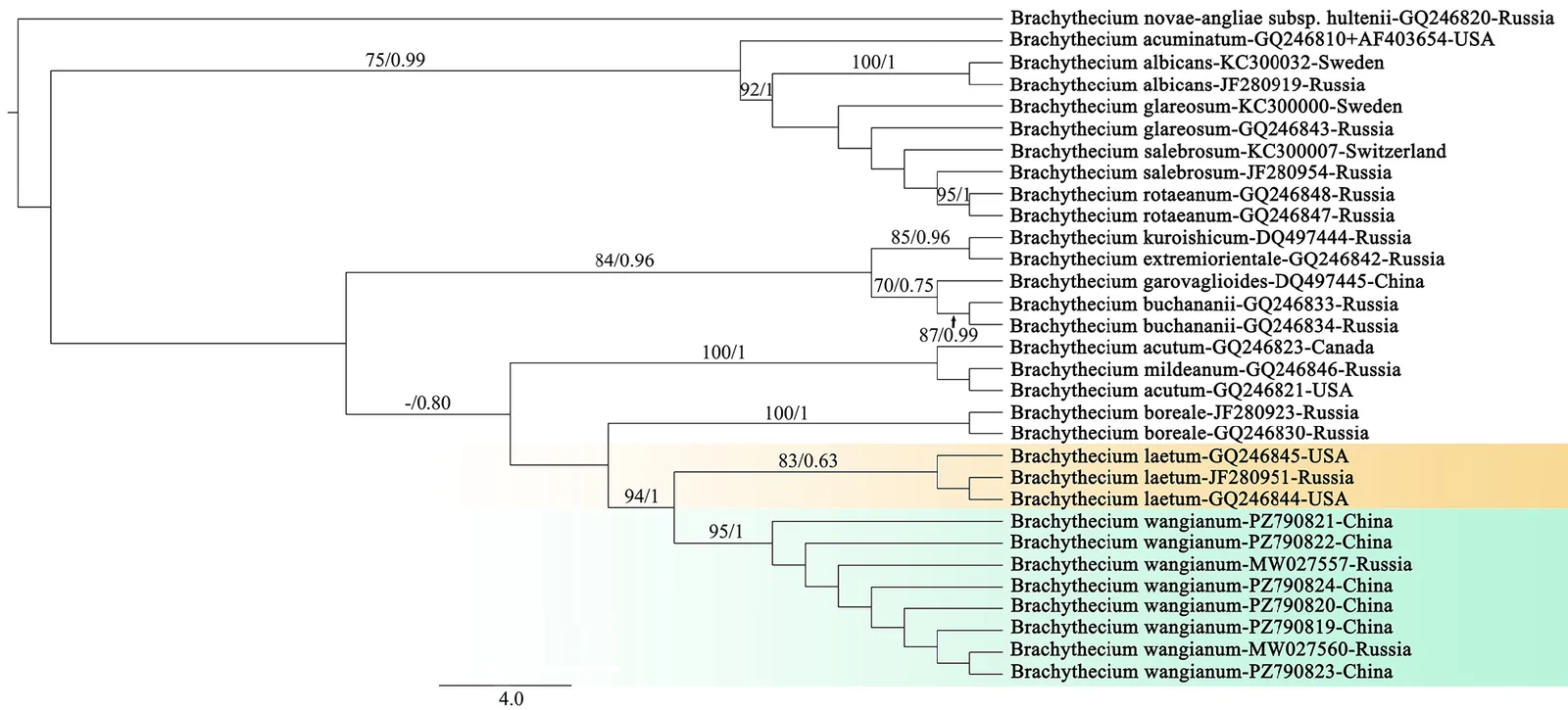
The phylogenetic tree from ML analysis with ITS dataset. Bayesian posterior probabilities (right) and ML boot-strap support (left) values are shown above branches.

Sequences generated in this study were aligned with those retrieved from GenBank (National Center for Biotechnology Information, NCBI; https://www.ncbi.nlm.nih.gov) (Table 1) using BioEdit v.7.0.8 (Hall 1999). Phylogenetic analyses were conducted using PhyloSuite v.1.2.3 (Zhang et al. 2020), following the analytical pipeline of Abdugeni et al. (2026). Both Bayesian Inference (BI) and Maximum Likelihood (ML) methods were employed. The final phylogenetic tree was reconstructed based on the ML topology, with branch support indicated by bootstrap values ≥ 70 and posterior probabilities ≥ 0.70 retained (Fig. 3).

**Table 1.** Specimens for which ITS sequences were obtained.

**Taxon**	**Collection No**.	**Locality**	**Herbarium**	**GenBank No**.
*Brachythecium novae-angliae* subsp. *hultenii*	*Ignatov 97-1282*	Russia	MHA	GQ246820
*Brachythecium acuminatum*	*Anderson 24257*	USA	H	GQ246810 + AF403654
*Brachythecium rotaeanum*	*Afonina 24.VIII.2001*	Russia	MHA ex LE	GQ246847
*Brachythecium rotaeanum*	*Suragina 5.V.2002*	Russia	MHA	GQ246848
*Brachythecium glareosum*	*Hedenäs S-B89992*	Sweden	S	KC300000
*Brachythecium glareosum*	*Ignatov14.V.1999*	Russia	MHA	GQ246843
*Brachythecium salebrosum*	*Hedenäs S-B6810*	Switzerland	S	KC300007
*Brachythecium salebrosum*	*Czernyadjeva #17*	Russia	LE ex MHA	JF280954
*Brachythecium albicans*	*Hedenäs S-B88283*	Sweden	S	KC300032
*Brachythecium albicans*	*Czernyadjeva 5*	Russia	MHA	JF280919
*Brachythecium kuroishicum*	*Cherdantseva 15.IV.1991*	Russia	MHA ex VLAD	DQ497444
*Brachythecium extremiorientale*	*Ignatov* & *Ignatova 06-2935*	Russia	MHA	GQ246842
*Brachythecium garovaglioides*	*Fuan 960785*	China	PE ex MHA	DQ497445
*Brachythecium buchananii*	*Ignatov 29/29*	Russia	MHA	GQ246833
*Brachythecium buchananii*	*Ignatov 06-196*	Russia	MHA	GQ246834
*Brachythecium acutum*	*Buck 52529*	Canada	NY	GQ246823
*Brachythecium acutum*	*Allen 28201*	USA	MO	GQ246821
*Brachythecium mildeanum*	*Ignatov* & *Ignatova 18.VIII.2003*	Russia	MHA	GQ246846
*Brachythecium boreale*	*Czernyadjeva 63*	Russia	LE ex MHA	JF280923
*Brachythecium boreale*	*Fedosov 05-650*	Russia	MW	GQ246830
*Brachythecium laetum*	*Allen 28393*	USA	MO	GQ246845
*Brachythecium laetum*	*Bersanova s.n*.	Russia	MHA	JF280951
*Brachythecium laetum*	*Buck 36983*	USA	NY	GQ246844
*Brachythecium wangianum*	*J. Kou 20200903049*	China	HBNU	PZ790819
*Brachythecium wangianum*	*M. Li 2015080805*	China	HBNU	PZ790820
*Brachythecium wangianum*	*M. Li* & *M. Sulayman 2024050413*	China	HBNU	PZ790821
*Brachythecium wangianum*	*M. Li 2014080301*	China	HBNU	PZ790822
*Brachythecium wangianum*	*M. Li* & *X.T. Peng L20250918-66*	China	HBNU	PZ790823
*Brachythecium wangianum*	*M. Li* & *Y.T. Wang 2024110502*	China	HBNU	PZ790824
*Brachythecium wangianum*	*Kučera 2a*	Russia	CBFS ex BRNM	MW027557
*Brachythecium wangianum*	*Kučera 5a*	Russia	CBFS	MW027560

### Geographical distribution analyses

**Figure 4.**
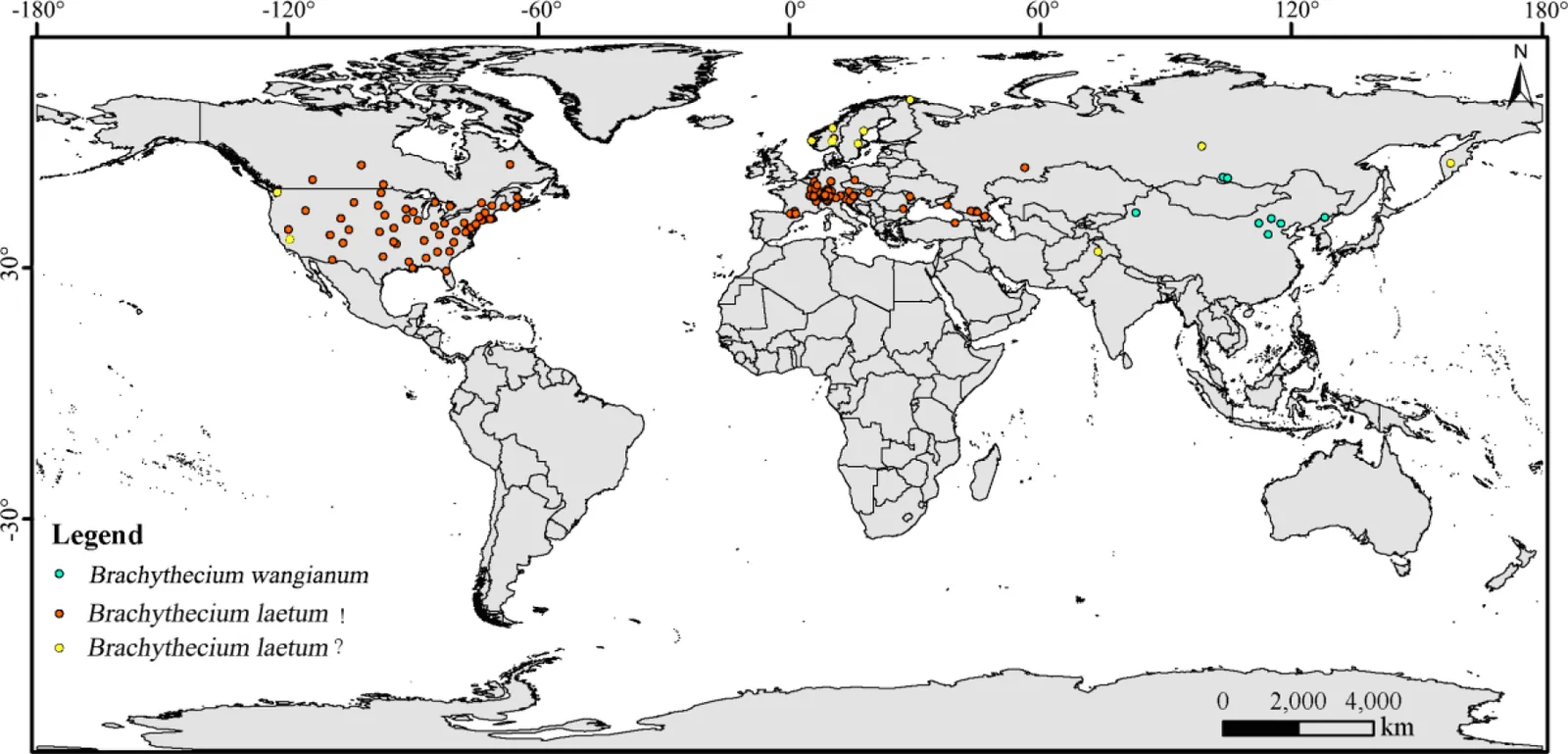
Global occurrence records of *Brachythecium wangianum* and *B. laetum*. Note: (!) Confirmed localities; (?) Unconfirmed, questionable distribution records.

This study documented eight distribution records for the new species, including six records from our own collections and two collections of Jan Kučera, published by Fedosov et al. (2022). A total of 4348 occurrence records for *Brachythecium laetum* were obtained from the Global Biodiversity Information Facility (GBIF.org (2026); http://www.gbif.org), Tropicos v.3.4.2 (https://www.tropicos.org) and relevant literature (Ignatov et al. 2008; Orgaz et al. 2012; Ignatov 2020). After filtering, 123 valid occurrence points were retained, with only one record per continent or province kept to avoid spatial clustering. The processed data were imported into ArcGIS 10.4.1 to generate distribution maps for the new species and its close relative *B. laetum* (Fig. 4).

The distribution of *Brachythecium laetum* is controversially treated in various publications. In North America, Ignatov (2014) outlined its absence in Pacific coastal states, following Persson (1947), Robinson (1962) and Lawton (1971), as well as the results of the revision of available herbarium collections. The GBIF distribution map (https://www.gbif.org/species/2679965, accessed 6 June 2026) indicates an abundance of localities in eastern North America, but also includes records from California and Washington States that require confirmation. However, the species reaches westwards to Nevada. Similarly, in Europe, this species is rare in the southern areas. Even in Spain, it was recorded for the first time only recently (Orgaz et al. 2012). Some GBIF records for Scandinavia are inconsistent with recent revisions of Hedenäs et al. (2014) and Hodgetts and Lockhart (2020). Therefore, records from areas where the species has never been observed by us are shown in yellow on the map.

## Results

### Phylogenetic analyses

Analysis of the nrITS dataset revealed that the Chinese specimens and Russian materials identified as *Brachythecium aff. laetum* (MW027557, MW027560) form a well-supported clade [PP = 1.00, BS = 95], which is sister to *B. laetum* (JF280951, GQ246844, GQ246845). The new species is further genetically distinct from the morphologically similar *B. salebrosum* and *B. rotaeanum* (Fig. 3).

### Geographical distribution analyses

*Brachythecium laetum* exhibits a disjunct distribution across the temperate zone of the Northern Hemisphere (Fig. 4), with two core areas in eastern North America and western Europe. Its range extends eastwards to Turkey and, in Russia, to the eastern Caucasus and South Urals. However, records from Pakistan further east remain unconfirmed. The species is absent from the Southern Hemisphere and tropical and subtropical low-latitude regions, being strictly confined to the temperate and boreal zones of the Northern Hemisphere.

### Taxonomic treatment

#### Brachythecium wangianum

Taxon classificationPlantaeHypnalesBrachytheciaceae

Min Li, X.T. Peng & Ignatov sp. nov.

317DC3B3-F985-5176-B2C7-76D848B6B7FF

Figs 1, 2

##### Diagnosis.

The species is most closely related to *Brachythecium laetum* phylogenetically, but can be distinguished morphologically from the latter by its longer and more slender leaf acumen, larger median laminal cells and an autoicous condition.

##### Type.

China • Hebei Province, Wuling Mountain National Nature Reserve, 40°35'17.01"N, 117°33'02.09"E, 907 m elev., 18 Sep. 2025, *M. Li & X.T. Peng L20250918-66* (holotype: HBNU!).

##### Description.

Plants medium-sized, 2–6 cm long, light green to yellowish green, slightly lustrous. Stems prostrate, with a central strand and a cortex of 3(–4) layers of incrassate cells, irregularly branched; branches robust, 5–15(–20) mm long, erect or slightly curved, terete when foliate. Proximal branch leaves triangular. Stem leaves erect when dry, loosely to densely inserted, (1.5–)2.0–3.0 × 0.7–1.0(–1.2) mm, ovate-lanceolate to broadly ovate-triangular, widest at 1/10–1/3 leaf length, gradually narrowed towards the apex, slightly constricted near the apex, acumen comprising 1/5–1/4 of total leaf length; leaves mostly concave, with one deep longitudinal plica on each side of costa extending to leaf margins; leaf margins plane or slightly recurved, denticulate; costa slender, extending to 1/2–2/3 leaf length, sometimes ending in a spine at apex; median laminal cells linear, (60–)70–100(–130) × 6–11 μm; leaf base rounded, not decurrent to slightly decurrent, margins plane or partially recurved, cells near base gradually shorter, slightly wider than median cells, 30–50(–65) × 8–15(–20) μm; alar cells thin-walled, subquadrate to oblong, 17–30(–50) × 15–20(–25) μm, arranged in 6–8 rows and together with the basal cells forming an indistinctly delimited, pellucid group. Branch leaves erect-spreading when dry, imbricate, lanceolate to ovate-lanceolate, (1.2–)1.5–2.6 × 0.4–0.75(–1.0) mm, concave, slightly plicate; margins serrulate; costa extending to 1/2–3/4 leaf length, usually ending in a spine; median laminal cells linear, smooth, 60–100(–130) × (6–)7–10 μm; leaf base not decurrent, basal cells rectangular to elliptical; alar cells quadrate to subquadrate, 15–30(–40) × 8–20(–25) μm.

##### Autoicous.

Perigonial leaves broadly ovate, with a single costa, apex acute or shortly acuminate; margins entire, bordered. Inner perichaetial leaves lanceolate, filiform-acuminate, with a single costa; outer perichaetial leaves shorter, ecostate. Seta reddish-brown, smooth, 0.5–1.5 cm long; capsule suberect to inclined, ovoid, curved and asymmetric; operculum long-conic; annulus differentiated, non-inflated, 1–2 rows of cells, fragmenting and deciduous; exostome teeth reddish brown, transversely striolate below, densely papillose above; endostome subequal to exostome in length, basal membrane 1/3–1/2 the height of exostome, cilia 1–3, distinctly nodose or protuberant; stomata phaneroporous, restricted to the capsule neck; spores ca. 14–20 μm in diameter, finely papillose.

##### Additional specimens examined

**(paratypes)**. China • Jilin, Baishan, Changbai Mountain, 02 Sep. 2020, *J. Kou 2020090233*, 03 Sep. 2020, *J. Kou 20200903049* (HBNU!). China • Hebei, Chengde, Wuling Mountain National Nature Reserve, 18 Sep. 2025, *M. Li & X.T. Peng L20250918-66* (HBNU!); • Shijiazhuang, Hebei Normal University, 05 Nov. 2024, *M. Li & Y.T. Wang 2024110502* (HBNU!); • Zhangjiakou, Tianlu Yanpian Mountain, 08 Aug. 2015, *M. Li 2015080805* (HBNU!). China • Inner Mongolia, Ulanqab, Erlongshitai National Forest Park, 03 Aug. 2014, *M. Li 2014080301*, *2014080343* (HBNU!). China • Xinjiang, Ili, West Tianshan National Nature Reserve, 04 May 2024, *M. Li & M. Sulayman 2024050413* (HBNU!).

##### Diagnostic features.

Leaves ovate-lanceolate to broadly ovate-triangular, distinctly concave; alar cells thin-walled, quadrate to subquadrate, not reaching the costa, together with the basal cells forming an indistinctly delimited, pellucid group; basal cells rectangular to elliptical, arranged in distinctly transverse rows; autoicous; seta smooth; annulus of 1–2 rows of non-inflated cells, fragmenting and deciduous.

##### Etymology.

The species epithet honorus Prof. Youfang Wang, an eminent bryologist who has long been dedicated to the taxonomic study of Brachytheciaceae in China. She compiled the Brachytheciaceae section in “Bryophytes of China” (Hu et al. 2008) and made important contributions to the research on this family.

##### Habitat and distribution.

*Brachythecium wangianum* grows on soil, humus or fallen logs in shaded habitats. Currently, it is known from northern China (Hebei, Inner Mongolia, Jilin, and Xinjiang) and in Baikal Lake area in southern Russia.

The species is currently known only from China and Russia. In China, it occurs disjunct in mountainous regions ranging from northeast China and north China to northwest China. In Russia, it grows on calcareous rocks within the mixed conifer and deciduous trees forests at low elevations. *Brachythecium wangianum* is absent from the core distribution ranges of *B. laetum* (e.g. eastern North America and western Europe), as well as from subtropical and tropical regions. Instead, its distribution is restricted to hemi-boreal to warm-temperate mountains of East and North Asia, producing a distinct pattern of geographical isolation and complementary distribution relative to *B. laetum*, which in Eurasia exhibits a strict western distribution.

## Discussion

Phylogenetic analyses (Fig. 3) confirm the placement of *Brachythecium wangianum* within *Brachythecium*. Its sequences cluster with other congeners of *Brachythecium* and form a sister clade to *B. laetum*, a placement corroborated by morphological features that align with the generic diagnostic characteristics.

*Brachythecium wangianum* groups with two Russian specimens identified as *B. aff. laetum* by Fedosov et al. (2022); (GenBank MW027557, MW027560) in a strongly supported clade, suggesting that the Chinese and Russian collections represent the same taxon.

*Brachythecium laetum* was originally described by Bridel (1827) in the genus *Hypnum* Hedw. and subsequently transferred to *Brachythecium* (Schimper, 1853). It was long treated as a synonym of *B. oxycladon* (Brid.) A. Jaeger (Grout 1897). Robinson and Ignatov (1997) re-examined the type specimens and confirmed *B. oxycladon* as conspecific with *B. acuminatum* (Hedw.) Austin, thereby restoring *B. laetum* as a distinct species.

Phylogenetic analyses place *B. wangianum* as sister to *B. laetum*, indicating a close relationship between the two species. Nevertheless, comparative morphological assessment shows that *B. wangianum* differs from *B. laetum* in several key diagnostic characters.

In overall leaf shape, the two species appear superficially similar. *Brachythecium wangianum* possesses a distinctly longer and more slender acumen, often comprising up to one-quarter of the total leaf length, and its leaves range from narrowly ovate-lanceolate to broadly ovate-lanceolate. In contrast, *B. laetum* has a shorter acumen and leaves that vary from narrowly ovate-triangular to ovate-triangular. In the latter species, prominent folds at the leaf base confer a rigid appearance, particularly in exposed sunny habitats. Under mesic and shaded conditions, however, its leaves develop a longer acumen and become superficially more similar to those of *B. wangianum*. Median laminal cells in *B. laetum* are linear and measure 25–70 × 5–8 μm, in contrast to the elongated-linear cells of *B. wangianum*, which measure (60–)70–100 (–130) × 6–11 μm and are significantly larger in both dimensions. With respect to sexual condition, Grout (1897) described *B. laetum* as dioicous with occasional monoicy. Robinson (1962) never observed autoicous plants in *B. laetum*, but repeatedly noted that the morphologically similar *B. rotaeanum* was “little more than an autoicous form of *B. oxycladon* (i.e. *B. laetum*)”. Subsequent studies of *B. rotaeanum* from different parts of North America and Eurasia using nrITS sequences, however, demonstrated that it is clearly distinct phylogenetically from *B. laetum* (Ignatov and Milyutina 2010). To our knowledge, no recent literature has reported a monoicous sexual condition in *B. laetum*, except *B. aff. laetum* from the Baikal area (Fedosov et al. 2022), as is *B. wangianum*. The occurrence of *B. aff. laetum* in nearby areas together with its full identity on ITS sequence supports referring these Russian plants to *B. wangianum*.

**Figure 5.**
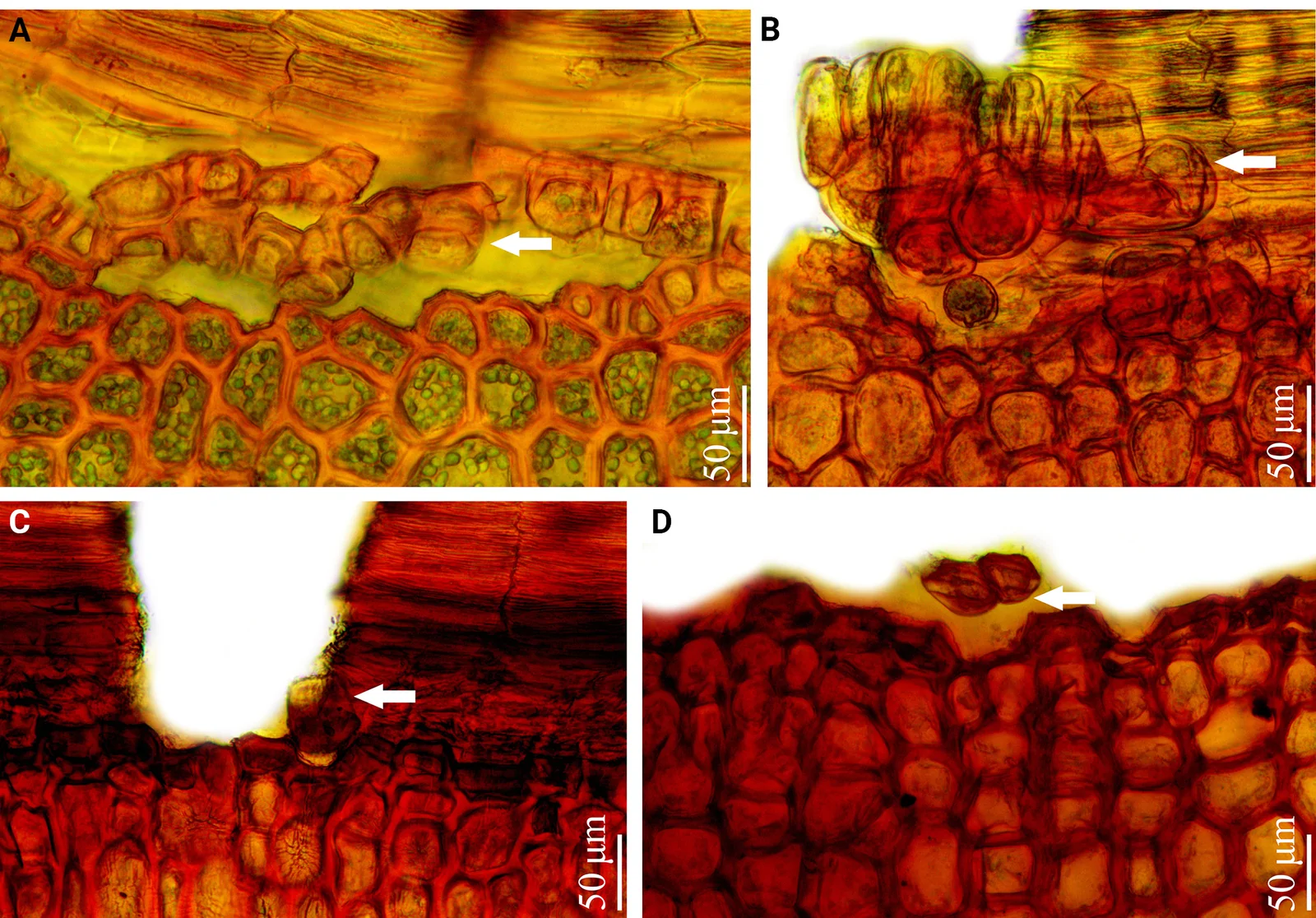
Microscopic features of the annulus. **A**. *Brachythecium wangianum*; **B**. *Brachythecium salebrosum*; **C, D**. *Brachythecium rotaeanum*.

*Brachythecium rotaeanum*, a common moss widely distributed across southern Siberia, closely resembles *B. wangianum* morphologically. The two species share a suite of morphological characters, including ovate-lanceolate stem leaves, elongate-linear median laminal cells up to 120 μm long, an indistinctly delimited pellucid group formed by alar and basal cells, autoicous sexual condition, smooth setae, and a differentiated caducous annulus of 1–2 rows of non-inflated cells (Fig. 5A, C, D). Despite these similarities, the two species can be reliably separated by several consistent diagnostic traits. The shoots of *B. rotaeanum* are julaceous and exhibit less regularly pinnate branching than those of *B. wangianum*. In addition, all alar cells of *B. rotaeanum* are non-pellucid, whereas some individuals of *B. wangianum* possess pellucid alar cells. A further diagnostic distinction is evident in capsule morphology: capsules of *B. rotaeanum* are only slightly curved to suberect, in contrast to the consistently curved capsules of *B. wangianum*.

Phylogenetically, the sister group to the clade comprising *B. wangianum* and *B. laetum* is *B. boreale* (Fig. 3), a widespread Siberian species that also occurs in north-western North America. It shares an autoicous sexual condition with *B. wangianum*, but differs in having mostly ovate leaves (similar to those shown in Fig. 1D: d). Since most leaves of *B. boreale* are of this shape, the species has a distinctly julaceous habit, markedly differing from that of *B. wangianum*. The alar cells of *B. boreale* are sparse and slightly pellucid; median laminal cells are 45–80 μm long, branches reach only 6 mm at most; and spores measure (18–)20–25 μm in diameter. These sparse, subpellucid alar cells occur in only a minority of *B. wangianum* specimens (e.g. Fig. 1N) and are totally absent in *B. laetum*. In contrast to *B. boreale*, *B. wangianum* shows variable leaf outlines and fully pellucid alar cells in some individuals, with much longer median laminal cells (60–)70–100(–130) μm, branches 5–15(–20) mm long, and smaller spores measuring 14–20 μm.

*Brachythecium wangianum* is also morphologically similar to *B. salebrosum*, with consistent characteristics in stem leaf morphology, costa length, median laminal cell shape, seta, capsule, operculum structure, and reproductive traits, but exhibits significant genetic divergence from the latter. Detailed examination revealed clear differences in branch length, alar cells and annulus. *B. salebrosum* has short branches, ca. 6–8 mm long, whereas *B. wangianum* has robust branches up to 5–15 (–20) mm long. Both species possess a caducous annulus composed of 1–2 cell rows, but the annulus of *B. salebrosum* is inflated, while that of *B. wangianum* is non-inflated (Fig. 5B). Additionally, *B. salebrosum* has a clearly delimited square group of small subquadrate cells, while *B. wangianum* has thin-walled, quadrate to subquadrate cells that form an indistinctly delimited, pellucid group with the basal cells. The latter serves as the principal diagnostic character states between the two species.

*Brachythecium falcatum*, originally described as *B. oxycladon* fo. falcatum (Grout 1928) and later synonymised under *B. salebrosum* (Crum and Anderson 1981), is superficially similar to *B. wangianum*, with which it shares a costa reaching 2/3–3/4 of the leaf length, long-linear upper laminal cells, smooth setae, and an autoicous sexual condition. Nevertheless, the two species are readily distinguishable by several stable morphological differences. In *B. falcatum*, stems and branches are somewhat curved at the ends and leaves strongly falcate-secund (Grout 1928; isotype at MICH)—features accounting for its specific epithet, in contrast to the straight stems and branches with erect-spreading leaves of *B. wangianum*. The alar cells are short-oblong, form a small, sharply delimited triangular cluster, whereas those of *B. wangianum* comprise thin-walled, subquadrate to oblong cells arranged in 6–8 rows, confluent with the basal cells to form an indistinctly bounded, pellucid group (Crum 2001). *B. falcatum* is also considerably smaller in overall dimensions: its leaves do not exceed 1.4 mm in length (Crum 2001), whereas those of *B. wangianum* are much larger. Furthermore, one side of the leaf margin is distinctly recurved along nearly the entire length, unlike the plane or only faintly recurved margins of *B. wangianum*. Its setae measure approximately 0.8 cm, shorter than those of *B. wangianum*. These consistent morphological differences clearly support the recognition of the two taxa as distinct species, although molecular evidence will be necessary to further clarify their phylogenetic relationships.

Based on integrated phylogenetic, morphological and biogeographical evidence, *B. wangianum* exhibits consistent and distinct differences from other related species in both molecular and morphological characters. Its discovery enriches the species diversity of *Brachythecium* in China and Russia. Furthermore, it provides new molecular and morphological data for phylogenetic studies of Brachytheciaceae and offers additional material for floristic and biogeographic research on temperate bryophytes.

### Key to *Brachythecium wangianum* and five closely related congeners

**Table:** 

1	Leaves ovate to ovate-lanceolate; alar cells larger than juxtacostal basal cells, or cells more or less evenly enlarged across leaf base	***B. boreale***
–	Leaves ovate-lanceolate to lanceolate, tapered; alar cells small, subquadrate to oblong–	**2**
2	Alar cells form a distinct opaque group, clearly demarcated from adjacent basal cells	**3**
–	Alar cells form a translucent or pellucid group, not clearly delimited from adjacent basal cells	**5**
3	Plants small; branch leaves falcate-secund and up to 1.4 mm long; setae to 0.8 cm long	***B. falcatum***
–	Plants medium-sized; branch leaves not falcate-secund and mostly exceeding 1.5 mm long; setae mostly longer than 0.8 cm	**4**
4	Median laminal cells up to 130 μm long; plicate on both sides of costa in stem leaves indistinct; autoicous; annulus fragmenting and deciduous	***B. salebrosum***
–	Median laminal cells up to 70 μm long; plicate on both sides of costa in stem leaves conspicuous; dioicous; annulus persistent	***B. laetum***
5	Shoots julaceous; branches to 8 mm long; alar cells always non-pellucid; capsules slightly curved or suberect	***B. rotaeanum***
–	Shoots not julaceous; branches 5–15(–20) mm long; some individuals with pellucid alar cells; capsules consistently curved	***B. wangianum***

## Supplementary Material

XML Treatment for Brachythecium wangianum

## Additional information

### Artificial Intelligence (AI) use

The authors accept full responsibility for the content of the manuscript, including the disclosure of any use of AI.

No AI tools were used in the preparation of this manuscript.

### Author ORCIDs

X.-T. Peng https://orcid.org/0009-0009-1910-4735

M.S. Ignatov https://orcid.org/0000-0001-6096-6315

Q.-N. Gu https://orcid.org/0009-0002-8596-031X

H.-Z. Liang https://orcid.org/0000-0002-7097-5941

C.-X. Chen https://orcid.org/0009-0000-3852-5716

Y.-T. Wang https://orcid.org/0000-0003-0508-332X

M. Li https://orcid.org/0000-0002-4025-6807

### Data availability

All of the data that support the findings of this study are available in the main text.
